# Are Irregularities of the Teeth Ever Caused by Tongue and Thumb Sucking?

**Published:** 1876-02

**Authors:** Jas. F. Thompson


### ARTICLE III.

### *Are Irregularities of the Teeth Ever Caused by Tongue and Thumb Sucking?*

BY JAS. F. THOMPSON, D. D. S., M. D.

This is a question I wish to present before the Virginia State Dental Association as a voluntary essay, and hope it will bring out considerable discussion. And since dental associations are organized for the purpose of discussing questions pertaining to the oral cavity, I therefore present this as a subject of itself, a very peculiar one, never having heard it or seen it discussed before by any dental association.

In answer to the question in the premises that I have propounded, I will say—yes; the most serious results are in a very large majority of cases produced by the causes I have named in the title. How often do we hear the old nurses of the little ones, whose existence in this world may be numbered by only a few days or a few weeks, say, "oh! let the little thing learn how to suck its thumb, it will make it so good." And then and there will commence a tuition that will result in what?—a deformity of the mouth and irregularity of the teeth that will cause many regrets in after life.

What is the normal type of the human jaw? It is round and parbolic. In the opposite of normal we find the jaw eliptical, contracted, disproportionately large and angular. This tpye we are apt to find in the case of those who have been addicted to the habit in question.

In the type of irregularity in this case, we find an undue prominence in the anterior incisive aspect of the superior maxillary, with much projection of the teeth, while the lower jaw in these cases is found so far as relates to its anterior aspects the reverse of the upper, being flattened while the teeth incline into the mouth, this being caused by the thumb or knuckles being pressed against the lower teeth, while the inside of the upper part of the thumb is brought against the back of the upper teeth, acting the part of a double lever. This mechanical distortion of the jaw causing irregularities of the teeth, has been one which has occupied my attention for several years past. I have observed it so closely that I have been able in almost every case to detect it.

I will cite a case in practice of a lady patient, aged 25 or 30, who came to me to have a partial dentine composing the upper front teeth inserted. Upon examining her mouth, and finding undue prominence of the anterior incisive portion of the superior maxillary, with the teeth of the lower jaw somewhat flattened in, I immediately asked her the question whether or not she was ever addicted to the habit of sucking her thumb when young. Without any hesitancy she told me she did so until she was ten or twelve years old, and when I pointed out to her how the bad formation of her mouth was brought about, she immediately commenced to censure those who had the care of her in her early childhood. Were it not for this deformity, she would be very good looking.

I have astonished mothers, who have come to me with their little six or seven year old ones, upon examining their mouths, by asking the question, if they had not been addicted to the habit of sucking their thumbs; andI invariably received an answer in the affirmative, and they would ask why should ask the question.

I would then point out my reason by calling their attention to the deformity, and irregularity of the teeth. They would be utterly surprised at the result, and would own that they had encouraged the habit in order to make them good. It is truly unfortunate that parents should be ignorant of the bad results that are brought about by this habit.

Sucking the tongue is another form which is very difficult to detect, and at the same time, the most serious of all, I believe it is a habit that is continued for a long time, and been run into adult age, and up to middle life we find some people constantly sucking their tongues when they get quiet, or on going to sleep or thinking about something. The tongue is then pressed like a stick against the upper jaw, and there constant pressure is made, so that persons who have this habit, have the jaw actually pushed out, not as it is with the thumb, but the whole arch pushed out.

I regret very much that I did not make plaster casts of the cases I have seen, which would show how exactly the thumb fits into the palate arch, and pushing the upper jaw out, and how the lower jaw seems to have been pushed in by the weight of the hand. These habits that I have spoken of, are apt to make some children weak, thin and pale, brought about no doubt by a constant flow of saliva, which is excited in the mouth by the act of sucking, causing a drain upon the system which is thrown into the stomach, thereby impairing the digestive apparatus as well as the appetite.

I have no doubt there are many cases where a dentist is at work trying to correct the evil of irregularities, while the patient is at work constantly producing it by the habits in question. Would it not be well for us as professional men, when we come across cases in our young patients like those I have enumerated, to investigate them through their parents, or those who have charge of them, and teach them how much injury is brought upon the little ones by the pernicious habit of sucking their thumbs? In fact it is a duty we owe to our patients and patrons, to tell them of these results so they can tell others.

In conclusion, I think I have beyond a question of doubt, shown how irregularities and deformities are produced by the habit of tongue and thumb sucking, and I do not know anything else which children do with their mouths that can deform them, except sucking.

